# Clinical manifestations and treatment strategies for congenital aural atresia with temporomandibular joint retroposition: a retrospective study of 30 patients

**DOI:** 10.1186/s40463-022-00615-4

**Published:** 2023-03-08

**Authors:** Lin Yang, Peiwei Chen, Yujie Liu, Jinsong Yang, Shouqin Zhao

**Affiliations:** Department of Otolaryngology Head and Neck Surgery, Beijing Tongren Hospital, Key Laboratory of Otolaryngology Head and Neck Surgery, Capital Medical University, Dongjiaominxinag No. 1, Dongcheng District, Beijing, 100730 China

**Keywords:** Congenital aural atresia, Temporomandibular joint, Manifestations, Congenital anomalies, External auditory canal

## Abstract

**Background:**

Patients with congenital aural atresia (CAA) can present with concomitant temporomandibular joint (TMJ) retroposition, implying that even with a high Jahrsdoerfer score, canaloplasty and tympanoplasty cannot be performed. Therefore, this study aimed to summarize the clinical manifestations and share our diagnostic and treatment experience of this rare entity, which has not been described previously.

**Methods:**

Thirty patients (30 ears) with CAA and TMJ retroposition without maxillofacial dysplasia were included. Diagnosis was based on patient history, physical examination, pure-tone average test results, and temporal bone high-resolution computed tomography (HRCT) findings. Their Jahrsdoerfer scores and interventions were also recorded.

**Results:**

Twenty-four and six patients among the 30 patients (males, n = 15) had CAA and TMJ retroposition on the right and left sides, respectively. Seventeen ears had a normal auricle; most had an enlarged cavum conchae and a large tragus. Twelve ears had an accessory auricle, and two had a preauricular fistula. All external auditory canals had complete atresia, including four with a shallow concavity and four with a small orifice in the cavum conchae. Temporal bone HRCT revealed poor or undeveloped tympanic temporal bone in the diseased ears, atresia in the external auditory canals, and partial/complete occupation of the mandibular condyle with or without soft tissue. The average Jahrsdoerfer score was 8.17. Thirteen patients opted for different surgeries, three wore a bone-conduction hearing aid, and fourteen chose no intervention.

**Conclusions:**

CAA with TMJ retroposition was often unilateral, typically on the right side. Most patients had normal auricles, with an enlarged cavum conchae and a large tragus (“mirror ear”). Even with a high Jahrsdoerfer score, traditional hearing reconstruction surgery could not be performed. Patients can undergo Vibrant Soundbridge or Bonebridge implantation or wear bone-conduction hearing aids to improve hearing levels, or refuse intervention because of mild hearing loss. The TMJ location can be used as a Jahrsdoerfer Grading System supplement for preoperative evaluation.

**Graphical Abstract:**

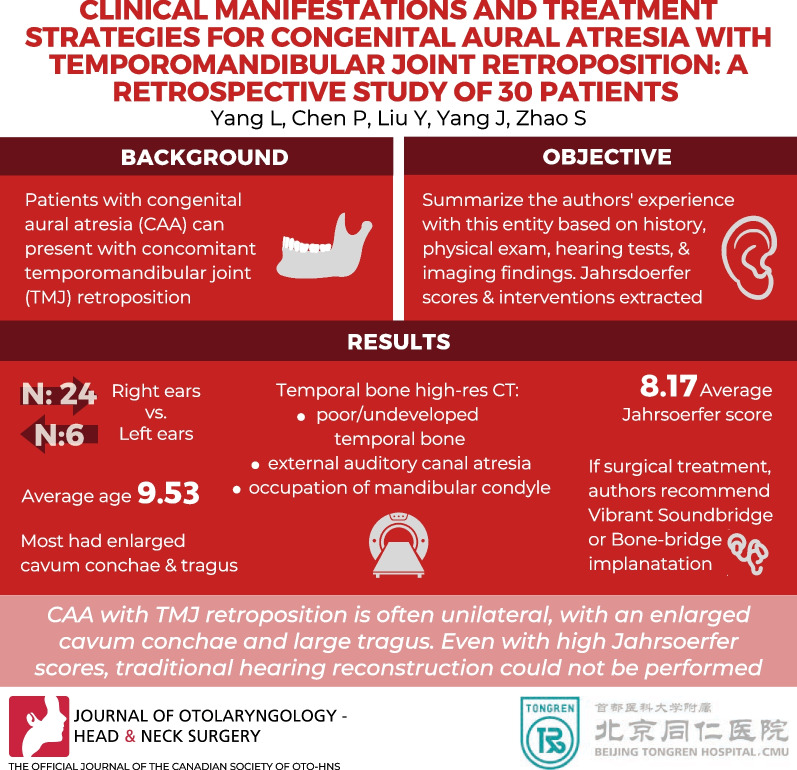

## Background

Congenital aural atresia (CAA) is an ear malformation evident at birth, with an incidence rate of 1:10,000–20,000 and a higher prevalence in men [1]. Most cases are associated with unilateral auricular deformities and middle ear malformations, commonly affecting the right side; however, the incidence of inner ear abnormalities remains relatively low. Additionally, some patients develop CAA as a component of different syndromes, including Treacher–Collins, Goldenhar, and Crouzon syndromes [2]. As the complex anatomical variations in CAA can lead to significant challenges in surgical repair, presurgical assessment is important. The Jahrsdoerfer Grading System is the most widely used preoperative evaluation method. However, surgical corrections—such as external auditory meatoplasty (canaloplasty) and tympanoplasty—are difficult to perform in patients with CAA and temporomandibular joint (TMJ) retroposition despite a Jahrsdoerfer score ≥ 6, as they may damage the TMJ [3, 4]. Therefore, in such patients, the choice of surgery should not be exclusively determined based on the Jahrsdoerfer Grading System. However, the clinical characteristics and treatment strategies of these patients remain unclear, and there are few relevant studies in this regard.

This study aimed to summarize the clinical manifestations of patients with CAA and TMJ retroposition without maxillofacial dysplasia or any syndromes to provide a reference for diagnosis. Furthermore, we aimed to share our clinical experiences regarding interventions for these patients.

## Methods

### Characteristics of participants

We retrospectively analyzed the clinical data of 30 patients (30 ears) diagnosed with CAA with TMJ retroposition at our hospital between April 2012 and August 2021. Patients with maxillofacial dysplasia were excluded from the study.

### Assessment of the degree of auricular deformity

All auricles were classified based on the severity of auricular deformity as Grades I, II, III, IV, and normal [1, 5]. In Grade I, the auricle was slightly smaller than normal, with mild deformities; however, each part could be clearly distinguished. In Grade II, the auricle was approximately one-half to two-thirds of the normal size with partial structure retention. In Grade III, the auricle was severely malformed and had a peanut or rope-like appearance, and in Grade IV, the auricle was absent.

### Pure-tone audiometry

Pure-tone audiometry (PTA) parameters were measured using a US GSI-61 (Ear Diagnostics Inc., Manila, Philippines) audiometer and analyzed. Bone conduction thresholds and air conduction thresholds were obtained at 0.25, 0.5, 1, 2, and 4 kHz. The air-bone gaps and average pure-tone threshold were calculated.

### Imaging evaluation

All patients underwent high-resolution computed tomography (HRCT) of the temporal bone in our hospital using a Philips Brilliance 64 CT scanner (Philips Medical Systems, Best, Netherlands) in a closed resting position. The imaging parameters were as follows: voltage, 120 kV; current, 200 mA; matrix, 512 × 512; and source image section thickness, 0.625 mm. Using a bone algorithm, the images were reconstructed in 1 mm slices in the axial, coronal, and sagittal planes. The window width was 4000 Hounsfield units (HU), and the window center was 700 HU. Two radiologists evaluated the external auditory canal (EAC), TMJ, and important middle and inner ear structures on HRCT images. An experienced otologist reviewed the cases, made a final diagnosis, and calculated the Jahrsdoerfer scores.

### Intervention

Interventions for individual patients were recorded based on the Jahrsdoerfer scores, our clinical experience, and the requirements of patients and their families.

## Results

### Patient characteristics

Of the enrolled participants, 15 were men and 15 were women. Twenty-four participants presented with right-sided CAA, six with left-sided CAA, and none with bilateral CAA. The maximum, minimum, and average ages were 30, 4, and 9.53 years, respectively (Table 1). Two ears had a preauricular fistula, wherein one ear had an inner ear malformation. Three patients had pathologies in their contralateral ear; of these, one had external auditory canal stenosis (EACS) with cholesteatoma, one had EACS without cholesteatoma, and one had atresia without TMJ retroposition.

**Table 1:** Partial clinical data of the 30 participants

No	Sex	Age (years)	Side	Severity of auricular deformity	Accessory auricle	EAC	Contralateral ear	Jahrsdoerfer scores	Intervention
1	M	6	R	(–)	Yes	Atresia	(–)	9	(–)
2	F	5	R	(–)	No	Atresia	(–)	7	(–)
3	M	6	R	Grade III	No	Atresia	EACS	7	Left side VSB implantation
4	M	5	R	Grade II	No	Atresia	(–)	7	(–)
5	M	5	R	Grade II	No	Atresia	(–)	8	(–)
6	M	6	R	(–)	No	Atresia	(–)	10	(–)
7	F	7	L	Grade I	No	Atresia with a shallow concavity	(–)	8	(–)
8	F	8	R	(–)	No	Atresia	CAA	9	Left side auricle reconstruction + BB implantation
9	F	7	R	(–)	No	Atresia	(–)	9	(–)
10	F	10	R	Grade III	No	Atresia	(–)	8	Right side auricle reconstruction
11	M	5	R	(–)	Yes	Atresia with a small orifice	(–)	8	(–)
12	F	12	R	Grade I	No	Atresia	(–)	8	(–)
13	F	7	R	(–)	No	Atresia with a small orifice	(–)	10	Wear a bone-conduction hearing aid
14	M	23	L	(–)	Yes	Atresia	(–)	9	(–)
15	F	6	R	(–)	Yes	Atresia with a shallow concavity	(–)	9	Right accessory auricle excision
16	M	7	R	Grade II	No	Atresia	(–)	9	(–)
17	F	6	R	Grade III	No	Atresia	(–)	7	Right side auricle reconstruction
18	M	17	R	(–)	Yes	Atresia	Accessory auricle	8	Bilateral side accessory auricle excision
19	F	19	R	(–)	Yes	Atresia	(–)	7	(–)
20	M	10	R	Grade II	No	Atresia	(–)	8	Right side auricle reconstruction + EAC canaloplasty + tympanoplasty
21	M	5	R	(–)	Yes	Atresia with a shallow concavity	Accessory auricle	7	Wear a bone-conduction hearing aid
22	F	30	L	(–)	No	Atresia	(–)	8	(–)
23	F	19	L	(–)	No	Atresia	EACS with cholesteatoma	7	Right side EAC canaloplasty + tympanoplasty
24	M	9	R	Grade III	No	Atresia	(–)	9	Right side auricle reconstruction
25	M	6	R	(–)	Yes	Atresia	Accessory auricle + preauricular flstula	7	Bilateral side accessory auricle + right side preauricular flstula excision
26	F	12	L	Grade I	Yes	Atresia with a shallow concavity	(–)	8	Left side accessory auricle excision
27	M	8	R	Grade II	Yes	Atresia with a small orifice	(–)	9	Right side auricle reconstruction + EAC canaloplasty + preauricular flstula excision
28	M	8	L	(–)	Yes	Atresia	(–)	9	Left side accessory auricle excision
29	F	10	R	Grade II	No	Atresia	(–)	7	(–)
30	F	4	R	(–)	Yes	Atresia with a small orifice	(–)	9	Wear a bone-conduction hearing aid

*M* male, *F* female, *L* left, *R* right, *B* bilateral, *(–)* normal or nonintervention, *CAA* congenital aural atresia, *TMJ* temporomandibular joint, *EAC* external auditory canal, *EACS* external auditory canal stenosis, *VSB* Vibrant Soundbridge, *BB* Bonebridge

### External ear morphology

**Fig. 1:**
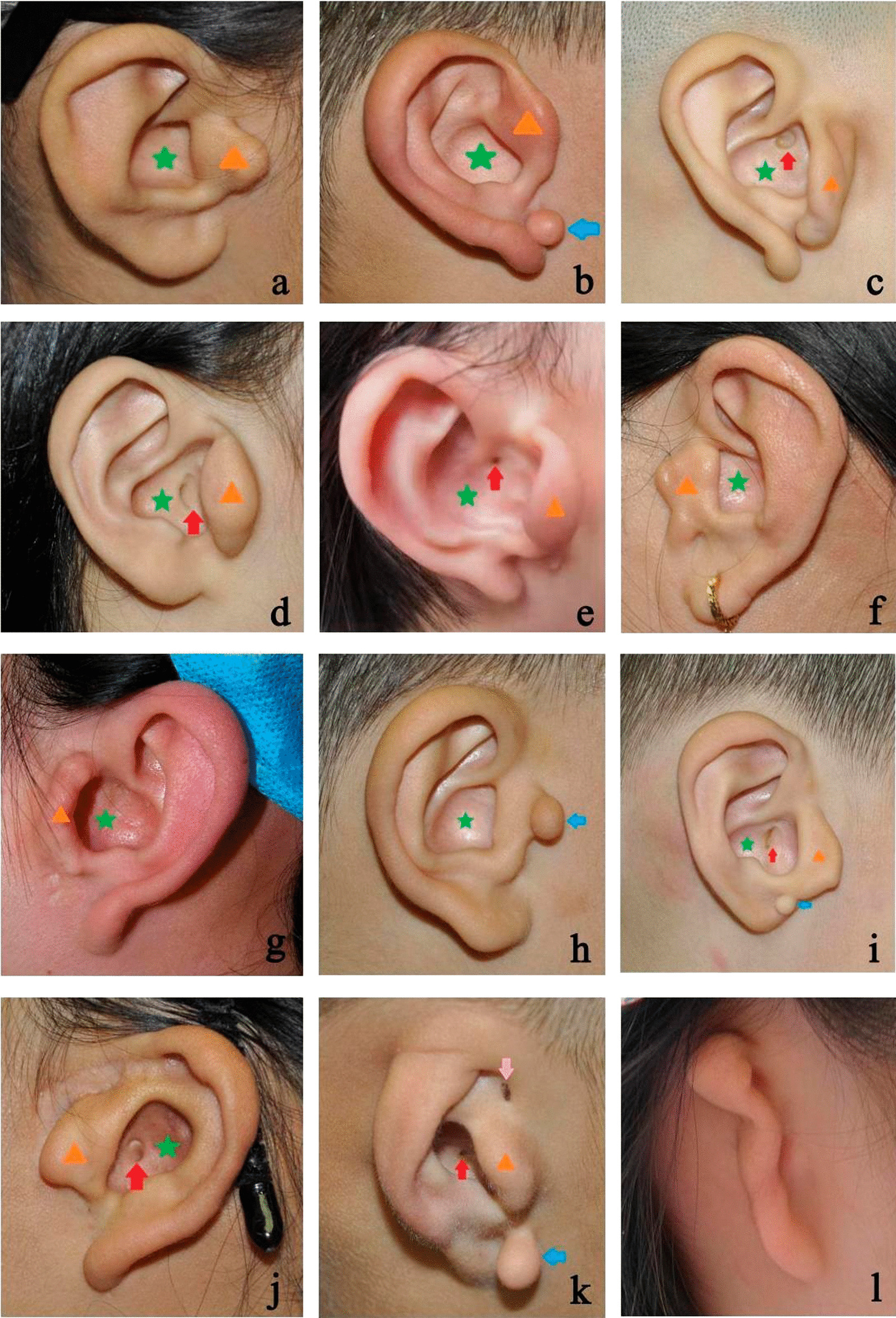
Morphological characteristics of the auricles. Star: enlarged cavum conchae; triangle: large tragus; left arrow: accessory auricle; downward arrow: preauricular fistula; upward arrow: shallow concavity (**c**, **j**), small orifice (**d**, **e**, **i**, **k**)

Of the 30 ears, 17 had a normal auricle shape, and most had an enlarged cavum conchae and a large tragus (Fig. 1a–i). Three ears were classified as Grade I (Fig. 1j); six as Grade II (Fig. 1k); four as Grade III (Fig. 1l); and none as Grade IV. Twelve ears had accessory auricles in the anterior region of the auricles (Fig. 1b, h, i, k). One patient had a 2-cm scar at the crus helix due to preauricular fistula resection (Fig. 1j), and two patients had a preauricular fistula at the crus helix without infection (Fig. 1k). All EACs were atresic; four patients had a shallow concavity (Fig. 1c, j), and four had a small orifice (Fig. 1d, e, i, k) in the cavum conchae (Table 1).

### Hearing results

The average bone conduction threshold was 6.30 ± 0.71 dBHL, the average air conduction threshold was 51.84 ± 9.19 dBHL, and the average air–bone gap was 45.53 ± 8.49 dBHL.

### Temporal bone high-resolution computed tomography

**Fig. 2:**
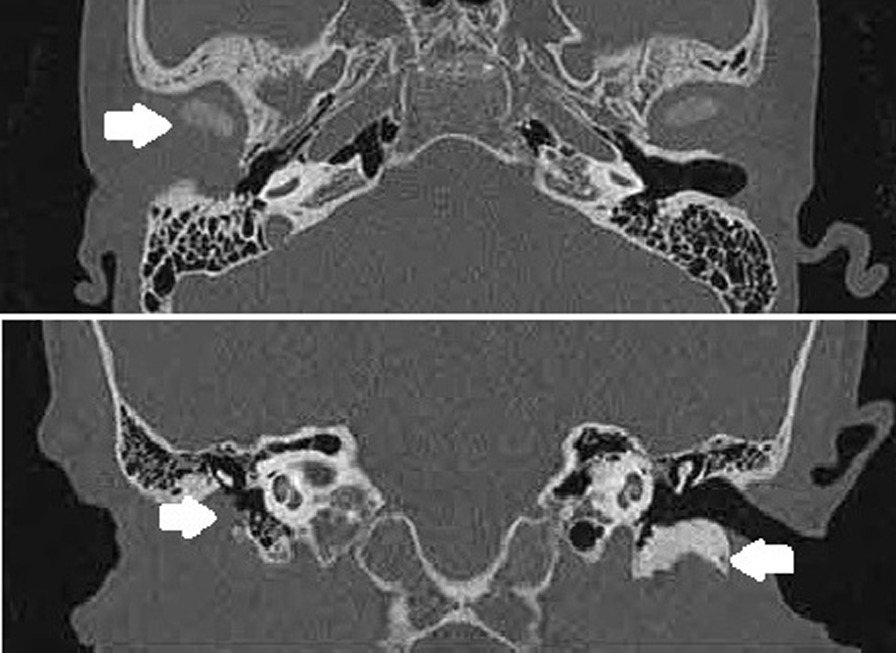
Temporal bone HRCT of patient No. 13 in the axial and coronal sections. Right arrow, undeveloped tympanic portion of the temporal bone and retrodisplaced mandibular condyle; left arrow, normal tympanic portion of the contralateral ear

Axial and coronal sections of the HRCT scan revealed a poorly developed or underdeveloped portion of the tympanic temporal bone. The lack of anterior and inferior EAC walls led to the occupation of the mandibular condyle in the EAC, sometimes with soft tissue deposition in the left space, referred to as TMJ retroposition (Fig. 2). The average Jahrsdoerfer score was 8.17 (Table 1).

### Intervention results

Of the 30 patients, one had auricular reconstruction, EAC canaloplasty, and tympanoplasty on the TMJ retroposition side; one had auricular reconstruction, EAC canaloplasty, and preauricular fistula excision on the TMJ retroposition side; three only had auricular reconstruction on the TMJ retroposition side; one had contralateral EAC canaloplasty and tympanoplasty; one had contralateral Vibrant Soundbridge (VSB) implantation; one had auricular reconstruction and Bonebridge (BB) implantation on the contralateral side; four had unilateral/bilateral excision of accessory auricles; one had bilateral excision of accessory auricles and deformity side preauricular fistula excision; three wore a bone-conduction hearing aid; and 14 chose no intervention (Table 1).

## Discussion

### Occurrence of congenital aural atresia with temporomandibular joint retroposition

Lumbroso et al. [6] reported posteriorly positioned TMJs in 16% of 67 cases of CAA/EACS. Moreover, Ren et al. [4] described the audiological features of 23 CAA patients with TMJ retroposition. These studies raised the question regarding the occurrence of CAA with TMJ retroposition.

Similarities in embryonic origin between the external and middle ear and the mandible can be affected by several factors during the development of these structures, causing abnormal auricular morphology, EAC stenosis or atresia, and mandibular deformity. The external and middle ear originate from the embryonal ectoderm layer, particularly the first pharyngeal cleft between the first and second pharyngeal arches [7–9]. The TMJ develops from the first pharyngeal arch [10]. However, the origin of the inner ear is different, which results in few accompanied inner ear malformations. Of the 30 participants in our study, only one had an inner ear deformity, which was a rare condition.

In addition, the EAC is anatomically adjacent to the TMJ, allowing them to potentially affect each other. The TMJ is usually located in front of the EAC, and they are separated by a bony anterior wall of variable thickness [11]. The anterior wall of the EAC consists of the tympanic bone. Several factors, such as trauma, tumors, injury, inflammation, and a patent foramen of Huschke, may cause protrusion of the TMJ into the EAC, leading to inflammation and stenosis of the EAC [12–14]. Meanwhile, some EAC diseases, such as cholesteatoma and malignancies, can directly destroy the anterior wall of the EAC and invade the TMJ [15–18].

Therefore, CAA and TMJ retroposition can occur simultaneously, accompanied by other external and middle ear malformations and, rarely, inner ear deformities. There may be unidentified factors influencing the normal formation of the auricles and EAC resulting in the termination or continuation of the process in a disordered fashion. For example, the tympanic part of the temporal bone could be poorly developed or undeveloped, causing variations in the anatomy of the EAC. In this process, the TMJ moves posteriorly and occupies the position that should have developed into the EAC, eventually resulting in CAA with TMJ retroposition.

However, the exact reasons for these changes remain unclear. Some studies have suggested that the tympanic ring, derived from the first pharyngeal arch, is significant for EAC formation [13, 18]; particularly, underdevelopment of the tympanic ring may result in EAC absence. This has been confirmed in gene-knockout experiments involving mice, which attracted attention to the relationship between the tympanic ring and EAC atresia in humans [18, 19]. Ozeki [8] observed that mice without the *ET-1* gene presented with auricular hypoplasia; this supports the association of the *ET-1* gene with auricle and EAC malformations. Nonetheless, further studies are required.

### Clinical manifestations of congenital aural atresia with temporomandibular joint retroposition

**Fig. 3:**
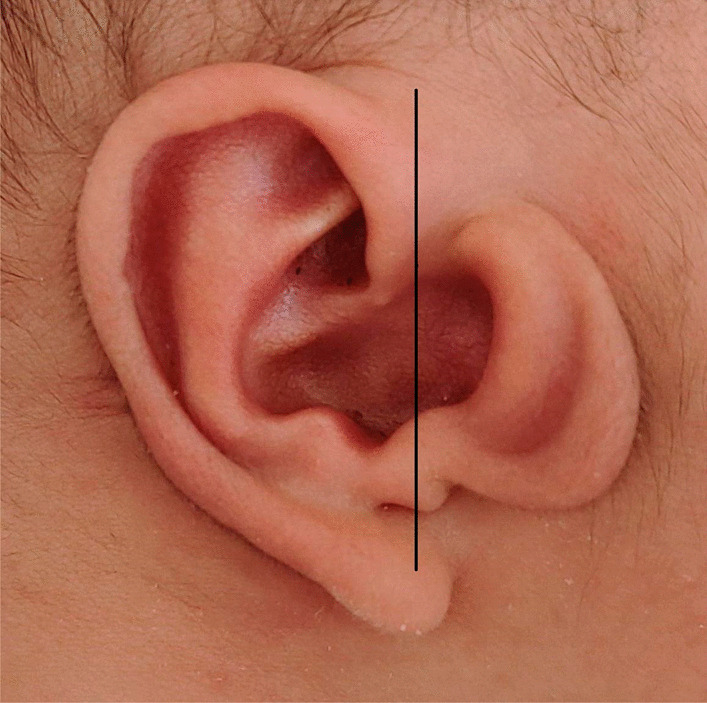
The “mirror ear”. The large tragus is like the shrunken and deformed image of the normal auricle in the mirror (the black line)

Based on our clinical observations, CAA with TMJ retroposition was often unilateral—mostly on the right side—and could not accompany by maxillofacial dysplasia or other symptoms. The auricles could be normal, slightly deformed, or cord-shaped. Nevertheless, most of the auricles were normal or had mild deformities and always had an enlarged cavum conchae and a large tragus. Additionally, some patients demonstrated accessory auricles and/or a preauricular fistula. The large tragus was an accessory auricle, and its shape was similar to that of a normal auricle but inverted, shrunken, and deformed, which we termed “mirror ear” (Fig. 3). When such findings are encountered, otologists should suspect CAA with TMJ retroposition. In our study, the bony EACs of all patients were either poorly developed or undeveloped and diagnosed as atresia. Most cartilaginous parts of the EACs were completely atretic; some had a shallow depression or a small orifice of different depths at the outer end of the undeveloped canal, with no history of suppuration, except in patient No. 27. The preauricular fistula of that patient was near the small orifice in the cavum conchae, with a history of watery discharge from the fistula orifice during a preauricular fistula infection.

In this study, less attention was paid to the results of hearing tests because we previously studied and reported the hearing characteristics of patients with this etiology [4]. We found that the conductive hearing loss of CAA with TMJ retroposition is milder than that without TMJ retroposition. This may be due to two reasons; first, there is a difference in sound energy loss between the bony atresia plate and TMJ, or second, the activity of the ossicular chain is better when it has a “soft connection” with the posterior TMJ than a “bone fusion” with the bony atresia plate. The unknown reasons are an interesting field for future research that requires a larger sample size.

The evaluation of temporal bone HRCT findings is important. It could help otologists determine the degree of deformity and choose a suitable strategy for hearing reconstruction. The most commonly used method of preoperative evaluation currently is the Jahrsdoerfer Grading System [20, 21]. CAA patients with a score ≥ 7 usually benefit more from EAC reconstruction and tympanoplasty than those with a score ≤ 6 [22]. However, the assessment of the TMJ location is not included in the grading system. In our study, all Jahrsdoerfer scores were higher than 7, which meant that the deformities of the CAA ears were mild and EAC canaloplasty and tympanoplasty could be performed. However, this traditional hearing reconstruction surgery could not be performed because the posterior TMJ occupied the EAC, and surgery could damage the TMJ and prevent the mouth from opening. Therefore, we suggest that the location of the TMJ should be added to the preoperative evaluation system as an important reference factor.

Hemifacial microsomia is the simultaneous occurrence of congenital micro-atresia (CMA) and mandibular hypoplasia [23, 24]. The first pharyngeal arch forms the maxillary and mandibular processes with a central cartilage inside; this is called Meckel’s cartilage and is related to mandibular development. The human Meckel’s cartilage reaches its full length in the 6th week and forms the tympanic and mandibular branches in the 16th week [25]. The end of the tympanic branch forms the incus and head of the malleus, and the mandibular branch is related to mandibular development. Therefore, CMA with hemifacial microsomia can coexist with mandibular malformations. However, TMJ abnormalities are relatively easy to diagnose in such patients. It is difficult to diagnose patients with CAA and TMJ retroposition without maxillofacial dysplasia. Moreover, this condition is frequently overlooked and misdiagnosed. This explains why we excluded patients with hemifacial microsomia from this study.

### Our treatment experience

Usually, CAA with TMJ retroposition can be diagnosed based on careful physical examination and HRCT findings. However, those with a shallow depression or a small orifice in the cavum conchae could be misdiagnosed as EACS with cholesteatoma, which should be distinguished [26].

The treatment objective includes improvement of esthetics and hearing. Auricular reconstruction and excision of accessory auricles and preauricular fistula could help to improve appearance. Hearing reconstruction methods are varied. In the early stage, due to our lack of experience with such diseases, we performed an EAC canaloplasty and tympanoplasty in patient No. 20 and an EAC canaloplasty in patient No. 27. We found that the operation was extremely difficult and the long-term hearing effect was not stable, despite short-term improvement following surgery. Therefore, we recommended VSB or BB implantation or the use of a bone-conduction hearing aid to improve hearing levels for future patients, even with a high Jahrsdoerfer score. However, some patients still chose observation and refused any treatment due to mild auricular deformity and hearing loss or the high price of hearing aids. Consequently, the final treatment strategy should be chosen based on both the wishes and economic conditions of patients and their families. Furthermore, some surgeries in the intervention were completed in stages, and the choice of hearing reconstruction method should consider the binaural condition.

There are some limitations to our study. We conducted a retrospective study of our clinical findings; therefore, data were limited. Moreover, the sample size was small because it focused on a rare entity. Future prospective cohort studies involving these patients should consider increasing the sample size and obtaining more reliable data.

## Conclusions

CAA with TMJ retroposition was often unilateral, typically on the right side. Most patients had a normal auricle with an enlarged cavum conchae and a large tragus (“mirror ear”). Although the Jahrsdoerfer score was high, traditional hearing reconstruction surgery (EAC canaloplasty and tympanoplasty) could not be performed. Patients could choose auricular reconstruction or repair to improve esthetics, receive VSB or BB implantation or wear bone-conduction hearing aids to improve hearing levels, or refuse intervention entirely because of the mild hearing loss. Given that the location of the TMJ affects the choice of hearing reconstruction method, we suggest this as an adjunct to the Jahrsdoerfer Grading System for preoperative evaluation.

## Data Availability

Not applicable.

## Abbreviations

CAA: Congenital aural atresia
TMJ: Temporomandibular joint
HRCT: High-resolution computed tomography
VSB: Vibrant Soundbridge
BB: Bonebridge
PTA: Pure-tone audiometry
HU: Hounsfield units
EAC: External auditory canal
EACS: External auditory canal stenosis
CMA: Congenital micro-atresia
